# Two new species of the tribe Hemisphaeriini (Hemiptera, Fulgoromorpha, Issidae) from southwestern China

**DOI:** 10.3897/zookeys.861.32594

**Published:** 2019-07-08

**Authors:** Liang-Jing Yang, Lin Yang, Zhi-Min Chang, Xiang-Sheng Chen

**Affiliations:** 1 Institute of Entomology, Guizhou University, Guiyang, Guizhou, 550025, China Guizhou University Guiyang China; 2 The Provincial Special Key Laboratory for Development and Utilization of Insect Resources, Guizhou University, Guiyang, Guizhou, 550025, China Liupanshui normal College Liupanshui China; 3 College of Animal Science, Guizhou University, Guiyang, Guizhou, 550025, China Guizhou University Guiyang China; 4 Office of Academic Affairs, Liupanshui normal College, Liupanshui, Guizhou 55300, China Liupanshui normal College Liupanshui China

**Keywords:** Fulgoroidea, morphology, Oriental region, planthoppers, taxonomy

## Abstract

Two new species of the tribe Hemisphaeriini: *Ceratogergithusbrachyspinus* Yang & Chen, **sp. nov.** (Yunnan) and *Neohemisphaeriusclavatus* Yang & Chen, **sp. nov.** (Guizhou) are described and illustrated. A checklist to Hemisphaeriini genera is provided. The generic characteristics of the genera *Ceratogergithus* Gnezdilov, 2017 and *Neohemisphaerius* Chen, Zhang & Chang, 2014 are redefined. Checklists and keys to the species of each genus are given.

## Introduction

Hemisphaeriini Melichar, 1906 is the second largest tribe of the planthopper family Issidae with currently 25 genera and 181 species known (Bourgoin 2018). It was erected by Melichar (1906) as family Hemisphaeridae but more recently Gnezdilov (2003, 2013a) downgraded it to the tribe level. Sun et al. (2015) raised the group again to the subfamily level based on partial sequences of the nuclear Wingless (Wg) and *18S rDNA* genes and Wang et al. (2016) enlarged the subfamily based on 18S, 28S, COXI and Cytb genes to include four tribes (Kodaianellini, Sarimini, Parahiraciini, Hemisphaeriini). However, here we prefer to follow Gnezdilov (2013a) and treat the group as a tribe of the subfamily Issinae which was also followed by Meng et al (2017).

Hemisphaeriini are characterized as follows: body hemispherical; vertex with anterior margin approximately transverse or triangularly elongate; pronotum with convex anterior margin; forewings thick and convex, claval suture present or absent, venation reticulate; hindwings single-lobed, being either well developed, i.e., longer than half length of forewings, venation reticulate, or rudimentary, shorter than half length of forewings, venation simple.

The tribe Hemisphaeriini is divided into two groups based on the presence or absence of the forewing claval suture. The genera *Neohemisphaerius* and *Paramongoliana* both have the forewing claval suture developed. The genus *Neohemisphaerius* was erected by Chen et al. (2014) for three species (*N.wugangensis*, *N.yangi* and *N.signifer* Walker, 1851) having a forewing with distinct claval suture. Recently Zhang et al. (2016) reviewed *Neohemisphaerius*, transferred species *N.signifer* Walker, 1851 to *Hemisphaerius* Schaum, 1850 and described species *N.guangxiensis* Zhang, Chang & Chen, 2016. The genus *Ceratogergithus* was erected by Gnezdilov (2017) for three species (*C.chelates*, *C.pseudotessellatus* and *C.spinosus*) having a forewing without a claval suture and pygofer with a large horn-shaped process on posterior margin. In this paper, we describe and illustrate two new species of the tribe Hemisphaeriini, give a checklist to Hemisphaeriini genera, redefine the generic characteristics and provided checklists and keys to the species of these two genera.

Hemisphaeriini are usually collected in broad-leaved forest, although some species are also found on Poaceae in open areas (Gnezdilov 2013b). The species *Neohemisphaeriusclavatus* Yang & Chen, sp. nov. was captured on *Bambusaemeiensis*.It maybe the second species that feeds exclusively on bamboos (host plant *Bambusaemeiensis*; Fig. 36), the other species is *Rotundiformanigrimaculata*, Meng, Wang & Qin, 2013, whose host plants may be *Gigantochloaligulata* Gamble and *Dendrocalamus* sp. (Meng, Wang & Qin, 2013).

## Materials and methods

The morphological terminology follows Chan and Yang (1994) and Bourgoin et al. (2015), except those for male genitalia following Gnezdilov (2003). Dry specimens were observed by stereoscopic microscope Leica M125 for illustration and description. All measurements are in millimeters (mm). The genital segments were separated and macerated in 10% NaOH, transferred to glycerine for observing and drawing. Illustrations of the specimens were made with a Leica MZ 12.5 stereomicroscope. Photographs of the types were taken by KEYENCE VHX-1000C.

The type specimens are deposited in the Institute of Entomology, Guizhou University, Guiyang, China (GUGC) and one paratype of *Neohemisphaeriusclavatus* Yang & Chen, sp. nov. in the Natural History Museum, London (BMNH).

### Checklist of genera of Hemisphaeriini

*Bolbosphaerius* Gnezdilov, 2013; Brunei, Vietnam.

*Bruneastrum* Gnezdilov, 2015; Borneo.

*Ceratogergithus* Gnezdilov, 2017; China: Hainan, Yunnan.

*Choutagus* Zhang, Wang & Che, 2006; China: Guangxi, Hainan.

*Clypeosmilus* Gnezdilov & A. Soulier-Perkins, 2017; Northern Vietnam.

*Euxaldar* Fennah, 1978; Vietnam.

*Epyhemisphaerius* Chan & Yang, 1994; China: Taiwan.

*Euhemisphaerius* Chan & Yang, 1994; China: Taiwan.

*Gergithus* Stål, 1870; India, Indonesia, Malaysia, Myanmar, Sri Lanka, Southern China, Thailand.

*Gergithoides* Schumacher, 1915; Japan, Southern China, Vietnam.

*Gnezdilovius* Meng, Webb & Wang, 2017; Southern China, Vietnam, Japan.

*Hemisphaerius* Schaum, 1850; China, India, Indonesia, Japan, Malaysia, Myanmar, New Guinea, Philippines, Sri Lanka, Thailand, Vietnam.

*Hemisphaeroides* Melichar, 1903; Sri Lanka.

*Hemiphile* Metcalf, 1952; Indonesia.

*Hysteropterissus* Melichar, 1906; New Guinea.

*Hysterosphaerius* Melichar, 1906; Singapore.

*Ishiharanus* Hori, 1969; Vietnam.

*Macrodaruma* Fennah, 1978; Southern China, Vietnam.

*Maculergithus* Constant & Pham, 2016; Northern Vietnam, Southern China.

*Mongoliana* Distant, 1906; Japan, Southern China.

*Neogergithoides* Sun, Meng & Wang, 2012; China: Guangxi, Guangdong, Hainan, Yunnan, Vienam.

*Neohemisphaerius* Chen, Zhang & Chang, 2014; Southern China.

*Ophthalmosphaerius* Gnezdilov, 2017; Southern China: Yunnan.

*Paramongoliana* Chen, Zhang & Chang, 2014; China: Guizhou.

*Rotundiforma* Meng, Wang & Qin, 2013; China: Yunnan.

## Taxonomy

### Family Issidae Spinola, 1839

#### Subfamily Issinae Spinola, 1839

##### Tribe Hemisphaerini Melichar, 1906

###### Genus Ceratogergithus

Taxon classificationAnimaliaHemipteraIssidae

Gnezdilov, 2017

####### Type species.

*Ceratgergithusspinosus* (Che, Zhang & Wang, 2007).

####### Diagnosis.

**Figures 1–4.**
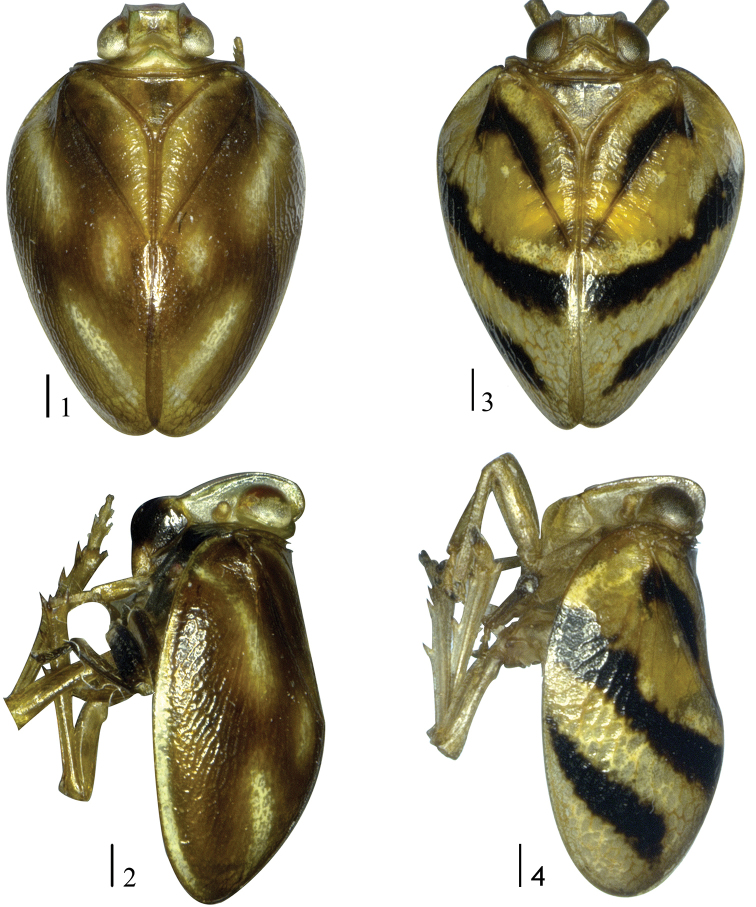
Dorsal and lateral habitus of two new species adult (male), **1, 2***Ceratogergithusbrachyspinus* Yang & Chen, sp. nov. **3, 4***Neohemisphaeriusclavatus* Yang & Chen, sp. nov. Scale bars: 0.5 mm.

Vertex subsquare or transverse. Metope wide, without median carinae. Postclypeus with distinct median carinae, elevated above the level surface of base of the frons (Figs 6, 7) or without carinae (Chen et al. 2014: figs 2–14D–E, 2–15D–E). Forewings without claval suture and shoulder-like projections (Chen et al. 2014: figs 2–14A–C, 2–15A–C) or with claval suture developed through its whole length, basally depressed (Figs 1, 2). Hindwing one lobed, longer than half length of forewing. Pygofer of male symmetrical (in lateral view), posterior margin with a large horn-shaped process in upper half. Anal tube of male apically enlarged (in dorsal view).

####### Distribution.

China: Hainan, Yunnan.

####### Discussion.

This genus is similar to *Gergithus* and *Neohemisphaerius*, but can be clearly separated from *Gergithus* by the posterior margin of the pygofer with a large horn-shaped process (Fig. 12) and the aedeagus without pair of short ventral directed toward its apex. It differs from the genus *Neohemisphaerius* by having a frons without a median carina, with colored marking, a hindwing well developed and longer than half the length of the forewing, and venation reticulate.

###### List of *Ceratogergithus* species

*Ceratogergithuschelates* (Che, Zhang & Wang, 2007); China: Hainan.

*Ceratogergithuspseudotessellatus* (Che, Zhang & Wang, 2007); China: Hainan.

*Ceratogergithusspinosus* (Che, Zhang & Wang, 2007); China: Hainan.

*Ceratogergithusbrachyspinus* Yang & Chen, sp. nov.; China: Yunnan.

###### Key to species of the genus *Ceratogergithus* (male)

**Table:** 

1	Clypeus with distinct median carina. Forewing with claval suture developed (Figs 6–8)	***C.brachyspinus* Yang & Chen, sp. nov.**
–	Clypeus without median carina. Forewing without claval suture	**2**
2	Forewing with four pale green transverse fasciae. Anal tube with apical margins strongly convex (in dorsal view) (Che et al. 2007: figs 26, 28)	***C.chelates* (Che, Zhang & Wang)**
–	Forewing and anal tube not as above	**3**
3	Forewing yellowish hazel. Anal tube with apical margins slightly concave (Che et al. 2007: figs 16, 18)	***C.spinosus* (Che, Zhang & Wang)**
–	Forewing dark with 3 large elongate spots in basal half, with 6 or 7 smaller elongate spots at apical margin in apical half. Anal tube margin nearly truncate (Che et al. 2007: figs 53, 55)	***C.pseudotessellatus* (Che, Zhang & Wang)**

####### Ceratogergithus brachyspinus

Taxon classificationAnimaliaHemipteraIssidae

Yang & Chen sp. nov.

http://zoobank.org/CA131906-B935-4958-8B37-BEB4B11CE542

Figs 1 , 2 , 5–18

######## Type material.

Holotype: ♂, China: **Yunnan**, Daweishan National Nature Reserve (103°20'E, 23°07'N), 8 May 2016, L.-J. Yang. Paratypes: 1♂, same data as holotype; 1♂, same data as holotype, except 19 August, 2017, Y.-J. Sui. All in GUGC.

######## Description.

Male body length (from apex of vertex to tip of forewing): 5.16–5.31 mm (n = 3); male forewing 4.43–4.58 mm (n = 3); male hindwing 3.30–3.47 mm (n = 3).

**Figures 5–19.**
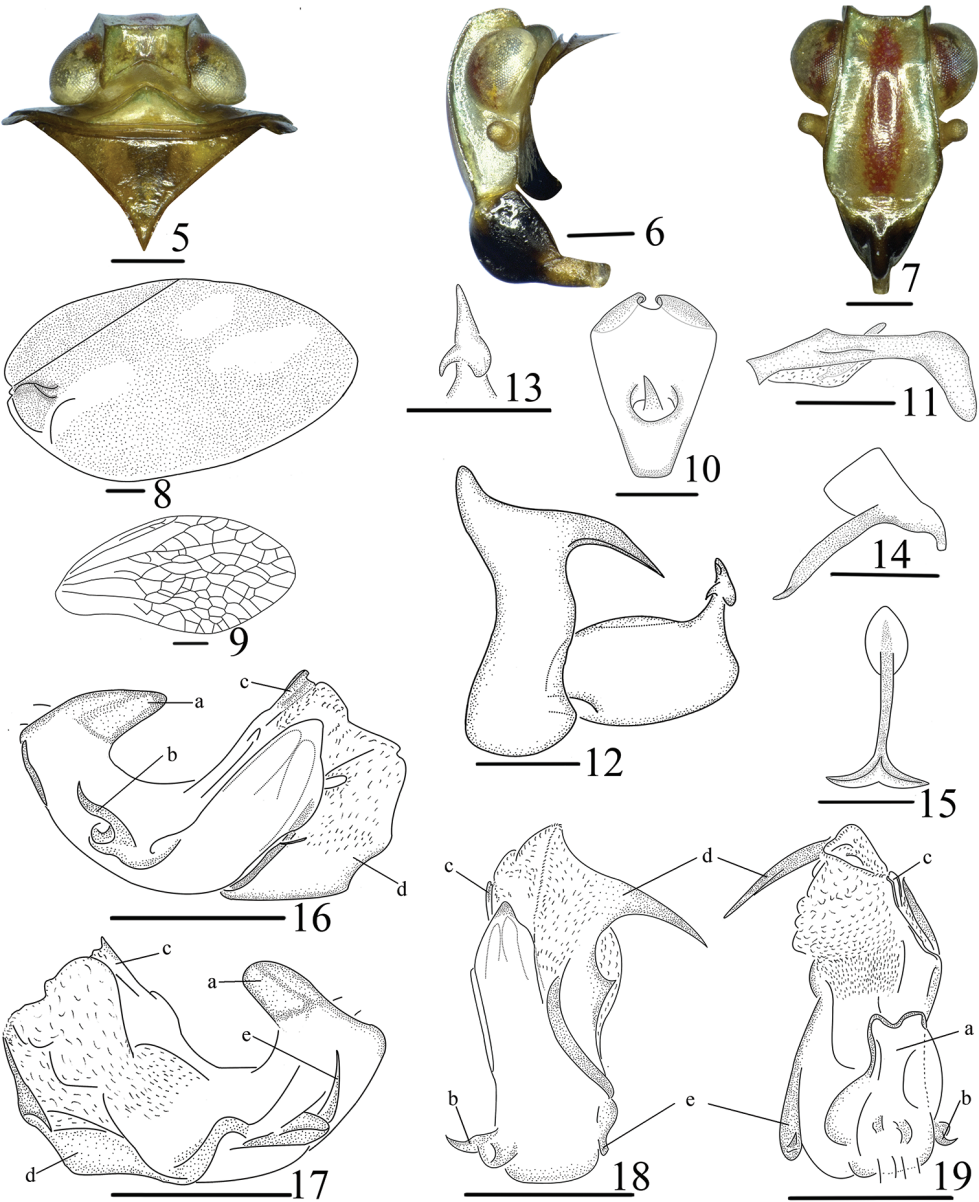
*Ceratogergithusbrachyspinus* Yang & Chen, sp. nov. adult (male), **5** head and thorax, dorsal view **6** head and thorax, lateral view **7** face, front view **8** fore wing **9** hindwings **10** anal tube, dorsal view **11** anal tube, lateral view **12** pygofer and genital style, lateral view **13** capitulum of gonostylus, dorsal view **14** connective, lateral view **15** connective, caudal view **16** penis, right lateral view **17** penis, left lateral view **18** penis, ventral view **19** penis, dorsal view. Scale bars: 0.5 mm.

**Coloration** (Figs 1, 2, 5–7). Vertex straw-yellow to pale green, all margins brownish (Fig. 5). Frons with brick-red markings, margins brownish (Fig. 7). Clypeus dark brown. Eyes reddish brown to greenish-brown (Figs 6, 7). Pronotum straw-yellow, margins brown (Fig. 5). Mesonotum (Fig. 5) fulvous, with fuscous subtriangular marking. Forewing fulvous, with three white markings irregular, costal margin white from middle to subapical part (Figs 1, 2, 8). Hindwing brownish and hyaline.

**Head and thorax** (Figs 5–9). Vertex shorter in middle than width at base (0.41: 1.00), transverse, anterior margin weakly convex, posterior margin angularly concave, disc depressed and all margins elevated (Figs 1, 5). Frons longer along midline than maximal width (1.53: 1.00) (Fig. 7), smooth, without median carina or pustules, apical margin nearly straight, margins carinate, disc slightly elevated (in frontal view) (Fig. 7) and arcuate (in lateral view) (Fig. 6). Clypeus with median carina obvious, postclypeus distinctly elevated (Figs 6, 7). Ocelli absent. Pronotum longer than vertex (1.56: 1.00), slightly depressed, margins elevated (Fig. 5). Mesonotum subtriangular, longer than pronotum (3.23:1.00) (Fig. 5), without median and lateral carinae, anterior margin nearly transverse (Fig. 5). Forewings about 2 times longer than maximal width (Figs 1, 2), with claval suture developed through its whole length, without “shoulder” basally, venation obscure. Hindwing 0.70 times as long as forewings (Figs 8, 9), reaching pygofer; venation reticulate (Fig. 9). Hind tibiae with two lateral teeth. Metatibiotarsal formula: 7–8–2.

**Male genitalia** (Figs 10–19). Anal tube 1.35 times as long as wide (in dorsal view) (Fig. 10), enlarged apically, apical margin deeply notched medially, bent ventrad (in lateral view) (Fig. 11). Pygofer symmetrical, posterior margin with large horn-shaped process in apical fourth (Fig. 12). Genital style subquadrate (in lateral view), moderately long, depressed in base near ventral margin, caudo-ventral angle rounded (Fig. 12). Capitulum with neck and small lateral tooth directed cephalad and big lateral tooth on posterior margin, directed laterad (Figs 12, 13). Connective cup-shaped (Figs 14, 15). Penis twisted medially (Figs 16, 17). Phallobase asymmetrical, with basal tooth process directed caudad (Figs 16, 17a), with pair of short lateral hooks in basal third, directed basad (Figs 16, 17b, e); dorso-lateral lobes of phallobase membranous in apical two-fifth (Figs 16, 19), with two differently shaped processes of different length directed apically: one process slender and short, arising in apical fourth (Figs 16–19c), other one arising in basal third, extended ventrad, with subapical process horn-shaped (Figs 17–19d). Ventral lobe of phallobase apically convex (in ventral view), shorter than dorso-lateral lobes (Fig. 18).

######## Etymology.

The specific name is derived from the Latin words “*brachys*”and “*spina*”, referring to the short lateral hooks on the basal third of the phallobase.

######## Host plant.

Unknown.

######## Distribution.

Southwestern China (Yunnan).

######## Remarks.

This species can be distinguished from all the other species of genus *Ceratogergithus* by the following characteristics: Frons with brick-red markings (Fig. 7); clypeus with distinct median carina, postclypeus distinctly elevated (Figs 6, 7); forewing fulvous, with three white irregular markings, with claval suture developed, basally depressed (Figs 1, 2).

####### Genus Neohemisphaerius

Taxon classificationAnimaliaHemipteraIssidae

Chen, Zhang & Chang, 2014

######## Type species.

*Neohemisphaeriuswugangensis* Chen, Zhang & Chang, 2014.

######## Diagnosis.

Body hemispherical. Vertex about 2.5–3.9 times as wider than long along midline, anterior margin straight, posterior margin angulately excavated. Frons elongate, with median carina, lateral margins elevated. Clypeus with median carina moderately convex, median carinae with or without a tubercle process in middle. Pronotum depressed, edges elevated. Mesonotum subtriangular, anterior margin approximately straight. Forewings hemispherical, claval suture developed, without shoulder-like projections basally. Hindwing rudimentary, shorter than half length of forewing, venation indistinct and simple. Hind tibiae with 2 lateral teeth. Metatibiotarsal formula: (9, 10)–(4, 5)–2. Anal tube of male wide and short. Phallobase with pair of ventral hooks directed basad.

######## Distribution.

China (Guangdong, Guangxi, Hunan, Guizhou).

######## Discussion.

*Neohemisphaerius* is similar to Hemisphaerius Schaum, 1850 and Gergithus Stål, 1870, but it differs from the two genera by having a frons with a median carina, and forewings with a claval suture developed. The genus *Neohemisphaerius* runs close to *Paramongoliana* in the key by Meng et al. (2017). It differs from *Paramongoliana* in: frons with median carinae, without a row of pustules along the lateral margins; clypeus distinctly convex on disc in midline; forewings with irregular markings; phallobase with pair of ventral hooks directed basad.

###### List of *Neohemisphaerius* species

*Neohemisphaeriusclavatus* Yang & Chen, sp. nov.; China: Guizhou.

*Neohemisphaeriusguangxiensis* Zhang, Chang & Chen, 2016; China: Guangxi.

*Neohemisphaeriuswugangensis* Chen, Zhang & Chang, 2014; China: Hunan.

*Neohemisphaeriusyangi* Chen, Zhang & Chang, 2014; China: Guangdong.

###### Key to species of the genus *Neohemisphaerius* (males; modified from Zhang et al. 2016)

**Table:** 

1	Frons with disc rugose (Fig. 22); clypeus with distinct median carinae (Fig. 22); forewings with three subparallel dark stripes, slanted caudad (Figs 3, 4); anal tube (in dorsal view) with apical margin concave medially (Fig. 25); Phallobase asymmetrical (Figs 33–35)	***N.clavatus* Yang & Chen, sp. nov.**
–	Frons with disc smooth (Zhang et al. 2016: figs 3, 16, 20); clypeus with a hump-shaped median carinae (Zhang et al. 2016: figs 2, 16, 19); anal tube not as above; phallobase symmetric	**2**
2	Forewings pale brown, with two black patches at costal margin (Zhang et al. 2016: figs 1, 2, 4–5); anal tube with apical margin medially convex (in dorsal view) (Zhang et al. 2016: fig. 12)	*** N. guangxiensis ***
–	Forewings black brown, with 4 or 5 light yellow patches (Zhang et al. 2016: figs 13, 14); anal tube not as above	**3**
3	Frons with obscurely short median carinae; anal tube with apical margin round (Chen et al. 2014: figs 2–36: H); a pair of ventral hooks of phallobase longer than half length of aedeagus	*** N. yangi ***
–	Frons with distinctly long median carina; apical margin of anal tube sinuate (in dorsal view) (Chen et al. 2014: fig. 2–35: H); a pair of ventral hooks of phallobase shorter than fifth length of aedeagus (Chen et al. 2014: figs 2–35: M, K)	*** N. wugangensis ***

####### Neohemisphaerius clavatus

Taxon classificationAnimaliaHemipteraIssidae

Yang & Chen sp. nov.

http://zoobank.org/DE9C89F6-24C8-4E2F-9EF1-E4354252141F

Figs 3 , 4 , 20–35 , 36

######## Type material.

Holotype: ♂, China: **Guizhou**, Duyun, Doupengshan (107°07'E, 25°51'N), L.-J. Yang, 19 August 2017; paratypes 2♂♂, same data as holotype; 3♂♂, same data as holotype except J.-K. Long, 8 August 2016. GUGC and one paratype in BMNH.

######## Description.

Male body length: 4.53–4.76 mm (n = 5); male forewings 4.23–4.38 mm (n = 5); male hindwing 1.17–1.42 (n = 5).

**Figures 20–35.**
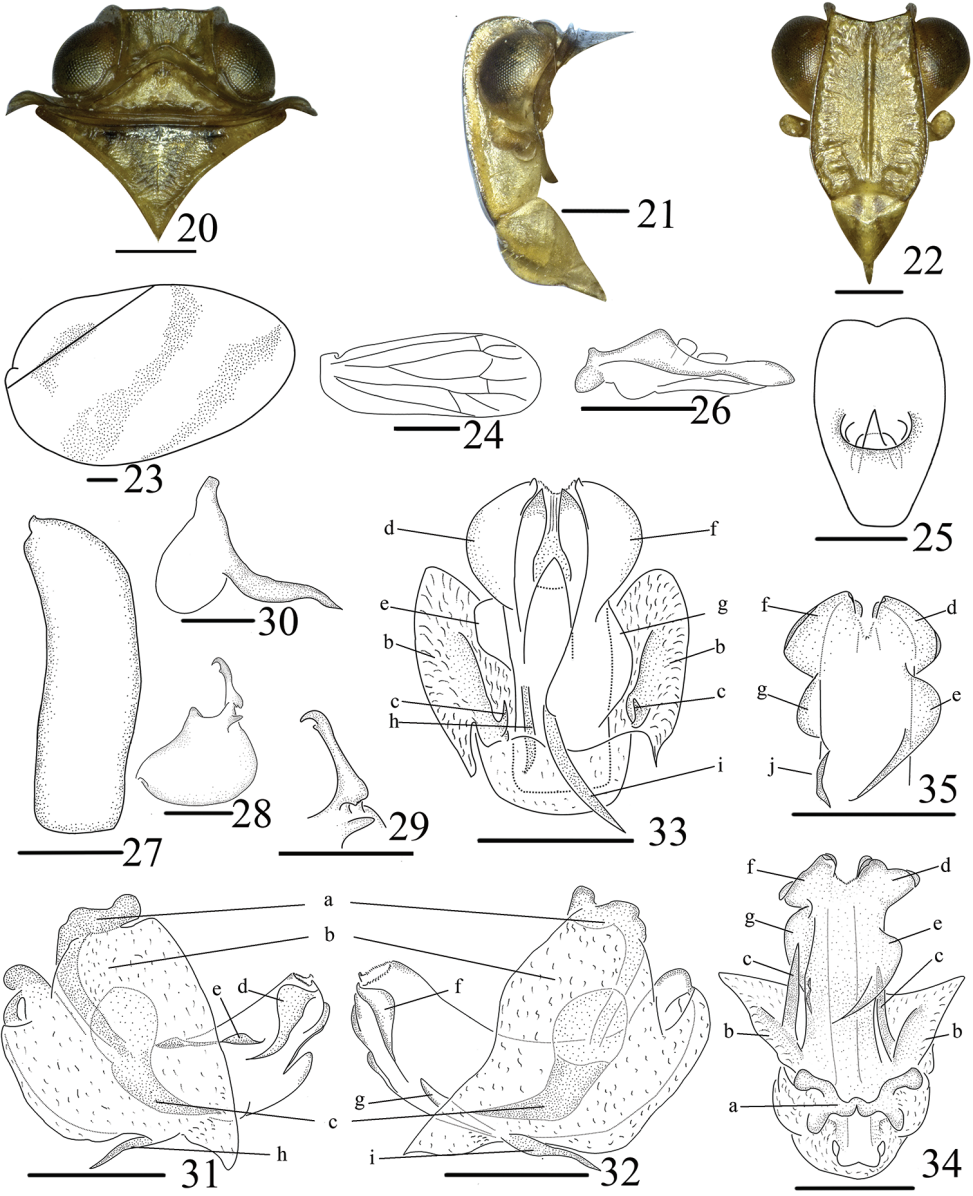
*Neohemisphaeriusclavatus* Yang & Chen, sp. nov. adult (male), **20** head and thorax, dorsal view **21** head and thorax, lateral view **22** head and thorax, front view **23** forewing **24** hindwing **25** anal tube, dorsal view **26** anal tube, lateral view **27** pygofer, lateral view **28** genital styles, lateral view **29** capitulum of gonostylus, dorsal view **30** connective, lateral view **31** penis left lateral view **32** penis, right lateral view **33** penis, ventral view **34** apical penis, dorsal view **35** penis, dorsal view. Scale bars: 0.5 mm.

**Coloration** (Figs 3, 4, 20–22). Head fulvous, margins of vertex and frons brown (Figs 20, 22). Clypeus with dark brown strip on each side of median carinae (Figs 21, 22). Rostrum brown (Figs 21, 22). Eyes dark brown, antennae brown (Fig. 21). Pronotum and mesonotum yellow brown, mesonotum with anterior margin dark brown in the middle (Fig. 20). Forewings yellowish and slightly pellucid, with three dark brown irregular stripes subparallel, slanted caudad, venation mostly fulvous (Figs 3, 4). Hindwing brownish hyaline. Legs brown. Abdomen yellowish.

**Head and thorax** (Figs 5–9). Vertex longer in middle than maximal width (0.37:1.00), quadrangular, anterior margin nearly straight, posterior margin angularly concave, margins elevated (Fig. 20). Frons rough, basally narrow, longer than maximal width in basal third (1.45:1.00), with median carinae, margins elevated (Figs 21–22). Clypeus with median carinae moderately convex, arcuate in lateral view (Figs 21–22). Pronotum longer than vertex in midline (1.63:1.00), slightly depressed, without carinae and pustules (Fig. 20). Mesonotum subtriangular, about 3 times longer than pronotum, anterior margin approximately straight (Fig. 20). Forewings about 1.70 times longer than maximal width, with claval suture developed through its whole, venation obscurely reticulate (Figs 3, 23). Hindwings rudimentary, shorter than half length of forewing, venation simple (Fig. 24). Hind tibiae with 2 lateral teeth. Metatibiotarsal formula of hind leg: 10–4–2.

**Male genitalia** (Figs 25–35). Anal tube pyriform, midline longer than broad (in dorsal view) (Fig. 25). Pygofer nearly rectangular (in lateral view), narrow, anterior and posterior margin subparallel (Fig. 27). Genital styles subtriangular (in lateral view), dorsal margin with triangular process, disc with fingerlike process below capitulum (Fig. 28). Capitulum with subapical tooth and lateral tooth (Figs 28, 29). Connective short and thick (Fig. 30). Phallobase asymmetrical, with process clavate, arched in basal third (in lateral view), directed basad, H-shaped (in dorsal view) (Figs 31, 32, 34a), process apically and phallobase basally with transparently membranous process with pair of strong hooks directed caudad (Figs 31–34b, c). Ventral lobe with pair of hooks asymmetrical in apical third, directed cephalad (Figs 31–34h, i); Lateral lobe bifurcate. Dorsal lobe with apical margin slightly notched medially (in dorsal view), with four differently sheet-shaped subapical processes (Figs 34–35d, e, f, g), the smallest near the left middle (Figs 34, 35g), with a short carinae left dorsally near its middle (Fig. 35j).

######## Etymology.

The name of new species is derived from the Latin words “clavate”, referring to the club-shaped process of the aedeagus in basal third (in lateral view).

######## Host plant.

*Bambusaemeiensis*.

######## Distribution.

Southwestern China (Guizhou).

######## Remarks.

This species resembles *N.wugangensis*, *N.yangi* and *N.guangxiensis*, but can be distinguished by the following characteristics: Frons rough (Fig. 22), disc flat, slightly depressed; clypeus with median carinae without a tubercles process in middle (Fig. 22); forewings yellowish brown, with three dark stripes subparallel (Figs 3, 4); anal tube with apical margin concave medially (in dorsal view) (Fig. 25); Phallobase asymmetrical, with process clavate in basal third (in lateral view), process directed basad, H-shaped (in dorsal view) (Figs 31, 32a, 34a).

**Figure 36.**
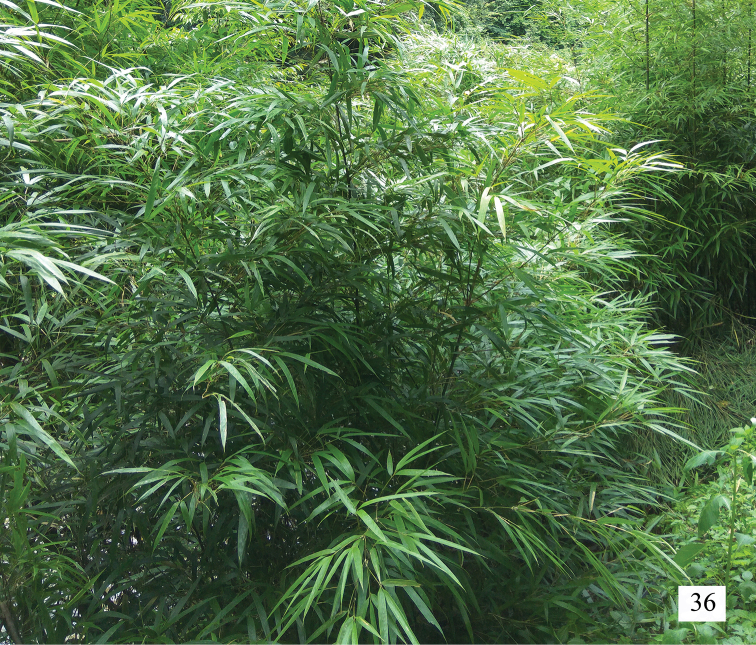
Host plant of *Neohemisphaeriusclavatus* Yang & Chen, sp. nov. in Doupengshan, Duyun (Guizhou, China). Photograph by L.-J. Yang.

## Supplementary Material

XML Treatment for Ceratogergithus

XML Treatment for Ceratogergithus brachyspinus

XML Treatment for Neohemisphaerius

XML Treatment for Neohemisphaerius clavatus
